# Phytochemical Analysis, Antioxidant and Antimicrobial Activities of *Helichrysum arenarium* (L.) Moench. and *Antennaria dioica* (L.) Gaertn. Flowers

**DOI:** 10.3390/molecules23020409

**Published:** 2018-02-13

**Authors:** Mihai Babotă, Andrei Mocan, Laurian Vlase, Ovidiu Crișan, Irina Ielciu, Ana-Maria Gheldiu, Dan Cristian Vodnar, Gianina Crișan, Ramona Păltinean

**Affiliations:** 1 Department of Pharmaceutical Botany, “Iuliu Hațieganu” University of Medicine and Pharmacy, 400337 Cluj-Napoca, Romania; babotamihai95@gmail.com (M.B.); mocan.andrei@umfcluj.ro (A.M.); irina.ielciu@umfcluj.ro (I.I.); gcrisan@umfcluj.ro (G.C.); rpaltinean@umfcluj.ro (R.P.); 2 Department of Pharmaceutical Technology and Biopharmaceutics, “Iuliu Hațieganu” University of Medicine and Pharmacy, 400010 Cluj-Napoca, Romania; gheldiu.ana@umfcluj.ro; 3 Department of Organic Chemistry, “Iuliu Hațieganu” University of Medicine and Pharmacy, 400010 Cluj-Napoca, Romania; 4 Department of Food Science, University of Agricultural Sciences and Veterinary Medicine, 400372 Cluj-Napoca, Romania; dan.vodnar@usamvcluj.ro

**Keywords:** *H. arenarium*, *A. dioica*, polyphenolic profile, LC-MS, antibacterial activity, antifungal activity, antioxidant capacity

## Abstract

*Antennaria dioica* (L.) Gaertn. and *Helichrysum arenarium* (L.) Moench. are two species of the Asteraceae family, known in Romanian traditional medicine for their diuretic, choleretic, and anti-inflammatory properties. The aim of the present study was to evaluate the phenolic and sterolic composition of flowers from the two species and to assess their antioxidant, antibacterial and antifungal properties. LC-MS analyses were performed on methanolic, ethanolic and 70% *v/v* ethanolic extracts, before and after acid hydrolysis, and revealed high amounts of polyphenols. Chlorogenic acid was found as the main compound for the flowers of *A. dioica* (502.70 ± 25.11 mg/100 g d.w.), while quercitrin was dominant in *H. arenarium* (424.28 ± 21.21 mg/100 g d.w.) in 70% *v*/*v* ethanolic extracts before hydrolysis. Antioxidant capacity assays showed an important antioxidant potential, which can be correlated with the determined polyphenolic compounds, showing the 70% *v*/*v* ethanolic extracts of the two species as being the most effective antioxidant samples for the DPPH assay. Antibacterial and antifungal assays confirm a modest biological potential for the same extract of both species. Results obtained in the present study bring important data and offer scientific evidence on the chemical composition and on the biological activities of the flowers belonging to the two species.

## 1. Introduction

The *Asteraceae* family, which comprises more than 1600 genera, with over 23,000 species, widespread in different types of climates and regions all over the world, is the largest family of flowering plants [1,2]. The diversity and heterogeneity of this family justifies the great importance of its individual members, which are known and used from ancient times, not only as food sources or as spices, but also for medicinal purposes [3,4]. Several classes of compounds from *Asteraceae* species were studied and tested for different bio-activities and were reported as having medicinal potential [3,4,5]. Among these compounds, a special attention has been given to polyphenols, and especially to flavonoids, which provide for these species important uses in the pharmaceutical, cosmetics and food industry, that are due to their important medicinal properties as the antioxidant, anti-inflammatory, antifungal or antibacterial ones [4]. In this context and taking into consideration the fact that in the last decades these compounds have shown a significant importance in the field of medicinal compounds, the *Asteraceae* species should be reconsidered as possible sources of flavonoids and polyphenols, generally.

**Figure 1:**
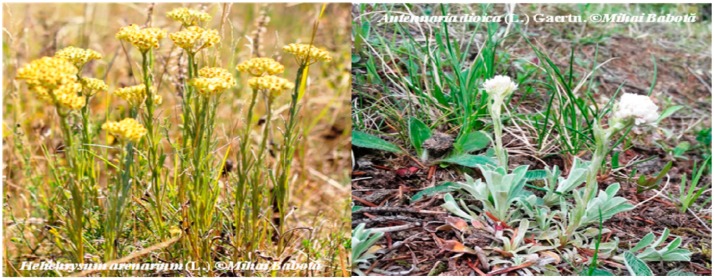
*Helichrysum arenarium* (L.) and *Antennaria dioica* (L.) Gaertn.

*Antennaria dioica* (L.) Gaertn. and *Helichrysum arenarium* (L.) Moench. (Figure 1) are two species belonging to the same tribe (*Gnaphalieae*) [6,7] of the *Asteraceae* family [8,9]. These species are widespread across the European continent, especially in Central and Eastern countries and are known in traditional medicine for their use in the treatment of different pathologies. The connection between these species relies on several common morphological characters and it is confirmed by phylogenetic studies [10,11], which certify their common taxonomic classification. The folk medicine of different countries cites common uses for herbal preparations obtained from the flowers of these species, exploited for their diuretic [12,13], choleretic and anti-inflammatory properties [13,14,15]. Romanian sources mention both under the same phytonyme, describing their use for the treatment of jaundice [16]. Few scientific evidence exists up to date in order to support these data and a more detailed study of the two species becomes therefore important.

*A. dioica* is a perennial herb, commonly found in dry grasslands and sandy or stony places from Eurasian areas, where it is traditionally used to treat biliary and respiratory ailments [13,15], and also for its astringent and hemostatic properties [13,17]. To date, few scientific data were reported regarding the chemical composition of this species. Meriҫli et al. [15] studied the extracts obtained from the flowers of *A. dioica* and isolated ursolic acid, chlorogenic acid, apigenin-7-*O*-glucoside and luteolin-7-*O*-glucoside. To the best of our knowledge, this is the only existing scientific study that describes the flavonoid composition of this species, and it does not establish a connection between the potential bioactivities of the species and the isolated compounds. 

*Helichrysum* species were known from ancient times due to a large distribution and diversity and are cited as being a potent remedy for various pathologies [12,14,18]. Among the species of this genus, a special importance was given to *H. arenarium*, which is the most popular species, having monographs in different pharmacopoeias, such as the Russian Pharmacopoeia [14]. Several studies aimed to investigate the phytochemical profile of *H. arenarium* and to evaluate its potential bio-activities [16,17,18,19,20]. Major constituents of the extracts obtained from the flowers of *H. arenarium* are polyphenols, especially flavonoids [19,20,21,22,23], usually found as glycosides. The connection between the phenolic content of this species and its antioxidant, anti-inflammatory and antimicrobial activities was studied by different authors [19,20,21]. Other studies focused on the investigation of the volatile compounds. In this regard, Rančić et al. [24] showed that the essential oil from *H. arenarium* flowers has an important antimicrobial potential. All existing data remain nevertheless scarce and do not establish a clear chemical composition of the flowers and their connection with the cited biological activities. 

Both species are used for different purposes in Romanian traditional medicine, but, despite this fact, few scientific data about the chemical composition and biological activities of *A. dioica* and *H. arenarium* collected from the Romanian spontaneous flora were reported so far. Grădinaru et al. investigated the phenolic content and antibacterial activity of a methanolic extract obtained from the inflorescences of *Helichrysum arenarium* (L.) Moench subsp. *arenarium* against lower respiratory tract pathogens, being the only existing scientific study about a Romanian *Helichrysum* sample [22]. Nonetheless, no scientific data with regard to Romanian *A. dioica* exist to date.

Within this frame, the present study aimed to investigate the phenolic, flavonoidic and sterolic composition of *A. dioica* (L.) Gaertn. and *H. arenarium* (L.) Moench. collected from the Romanian spontaneous flora and to assess their antioxidant and antimicrobial properties. Thus, the present study represents a starting point for a most detailed study of the two species, demonstrating their important potential as sources of bioactive compounds.

## 2. Results and Discussion

### 2.1. HPLC-MS Analysis of Polyphenolic Compounds

Qualitative and quantitative evaluation of phenolic compounds in the composition of the methanolic (MeOH), ethanolic (EtOH) and 70% (*v*/*v*) ethanolic (EtOH 70%) extracts obtained from the flowers of *A. dioica* and *H. arenarium* was achieved using an HPLC-MS method. The method was previously applied for the phytochemical analysis of different plant samples and provided good results [25,26]. Each sample was analyzed before and after acid hydrolysis in order to obtain more accurate data on the composition in flavonoid glycosides and their aglycones [26].

Among the 19 phenolic compounds used as standards, 9 compounds could be detected and quantified in hydrolyzed samples and 7 in non-hydrolyzed ones (Table 1 and Table 2). The external standard method was used for quantification, and results were expressed as mg compound/100 g dry weight (d.w.) herbal material.

Before hydrolysis, the sample that proved to contain the largest number of polyphenolic compounds was the 70% (*v*/*v*) ethanolic extract of the flowers of *H. arenarium*. In this sample, chlorogenic acid (340.95 ± 17.04/100 g d.w.) and quercitrin (424.28 ± 21.21 mg/100 g d.w.) were found in the highest amounts. High amounts of quercitrin were also found in the methanolic (438.33 ± 21.91 mg/100 g d.w) and ethanolic (409.09 ± 20.45 mg/100 g d.w.) extracts of this species. 

Similar results were obtained for the non-hydrolyzed extracts of *A. dioica*. Before hydrolysis, the most abundant compounds were chlorogenic acid (502.70 ± 25.11 mg/100 g d.w.) for the 70% (*v*/*v*) ethanolic extract, while for the methanolic and ethanolic extracts quercitrin was determined in the highest amount (methanolic extract: 444.48 ± 22.22 mg/100 g d.w., ethanolic extract: 147.64 ± 7.38 mg/100 g d.w.). Chlorogenic acid was also found in a significant amount in the methanolic extract (434.18 ± 21.70 mg/100 g d.w.).

After the acid hydrolysis, the presence of quercetol was determined in all samples, the 70% (*v*/*v*) ethanolic extract of *H. arenarium* containing the highest amount of this compound (26.16 ± 1.30 mg/100 g d.w.). A possible explanation of this fact is correlated with the decreasing amounts of isoquercitrin and quercitrin which partially hydrolyse into quercetol.

Our results indicate that major phenolic compounds of *H. arenarium* extracts are flavonoids (i.e., quercitrin, isoquercitrin). Other studies concerning the polyphenolic profile of this species were performed by Albayrak et al. [21] and Grinev et al. [23]. Their results show important amounts of phenolic compounds that are proved to be the main compounds in the extracts of this species (e.g., chlorogenic acid, quercetol, apigenin). Furthermore, using an HPLC-DAD-ESI-MS method, Grădinaru et al. [22] identified apigenin, kaempferol, chlorogenic acid, and caffeic acid in the composition of a methanolic extract obtained from *H. arenarium* ssp. *arenarium* flowers. The assays performed by the present study confirm the results obtained by these authors. However, this study brings novelty and originality, being the first report about the composition in phenolic compounds, such as *p*-coumaric acid, isoquercitrin, quercitrin and luteolin in the flowers of the Romanian *H. arenarium*.

Regarding the phenolic profile of *A dioica,* our results correlate with the previous report of Meriҫli et al. [15], which described the presence of chlorogenic acid, luteolin and apigenin, while providing, at the same time, new data on the presence of kaempferol (in the largest amount in the methanolic extract: 45.67 ± 2.28 mg/100 g d.w.) and quercitrin (in the largest amount in the methanolic extract: 444.48 ± 22.22 mg/100 g d.w.) in the non-hydrolyzed samples. Luteolin could be identified only in hydrolyzed samples, being found in the highest amount in the 70% ethanolic extract (183.52 ± 9.17 mg/100 g d.w.), together with *p*-coumaric acid (13.13 ± 0.65 mg/100 g d.w.), quercetol (19.66 ± 0.98 mg/100 g d.w.) and quercitrin (89.65 ± 4.48 mg/100 g d.w.).

### 2.2. HPLC-MS Analysis of Methoxylated Flavones

Among the tested methoxylated flavones, only hispidulin could be determined. Amounts that have been quantified do not exceed 1 mg/100 g d.w. The sample that proved to contain the highest amount of hispidulin was the methanolic extract of *H. arenarium* (0.70 ± 0.03 mg/100 g d.w.). Generally, this species proved to contain the largest amounts of hispidulin, while the amounts that are found in *A. dioica* are much lower (Table 3). This is the first report that assesses the amounts of methoxylated flavones among the components of the two species. 

### 2.3. HPLC-MS Analysis of Phytosterols

Five phytosterols were analyzed by an HPLC-MS method in the composition of the flowers belonging to the two species. Among these compounds, the highest amounts were found for β-sitosterol. Methanolic extracts of the flowers proved to be the richest samples in this compound: 37.04 ± 1.85 mg/100 g d.w. for *H. arenarium*, and 63.77 ± 3.18 mg/100 g d.w. for *A. dioica*. Lower amounts were found for stigmasterol and campesterol (Table 4).

### 2.4. Antioxidant Activity Assays

#### 2.4.1. Trolox Equivalents Antioxidant Capacity (TEAC) Assay

The TEAC assay is based on electron transfer reactions to evaluate radical scavenging activity of various compounds or complex samples. The antioxidant activity against the stable synthetic ABTS radical cation of the different *H. arenarium* and *A. dioica* extracts is summarized in Table 5. Some differences have been found in the antioxidant activities of the samples. The highest antioxidant activity was found for the 70% (*v*/*v*) ethanolic extract of the flowers belonging to both species (5.71 TE/mL and 5.82 TE/mL extract, for *A. dioica* and *H. arenarium*, respectively) and the lowest in the absolute ethanol-mediated extraction samples (2.65 TE/mL and 3.71 TE/mL). Previous data concerning the antioxidant activity of *H. arenarium* were obtained by Kiselova et al. [27] using the TEAC assay on Bulgarian *H. arenarium*. These authors indicated that aqueous extract of the investigated species possess a value of 1.50 ± 0.06 mM TE. Nevertheless, it is not possible to compare the present results with those from the literature, because of the different ways of expression, and/or the different preparation method/type of sample.

#### 2.4.2. DPPH Assay

DPPH is a stable free stable radical and its absorbance maximum occurs at 517 nm. The higher the rate of DPPH consumption is, the more powerful the antioxidant potential. The results obtained from the DPPH assay were presented as Trolox equivalents (Table 5). The values obtained for *A. dioica* are in accordance with the values obtained from TEAC assay. The 70% (*v*/*v*) ethanolic extract possessed the highest value (15.21 mg TE/mL), whereas the absolute ethanolic extract showed a value of 5.89 mg TE/mL. There are few available data regarding the antioxidant capacity of *Antennaria* species. In this case, a comparison with the results of other researchers is not possible. In contrast to this, there are various studies regarding the antioxidant proprieties of *H. arenarium*. Albayrak et al. [28] indicated that the extract of the investigated species possessed an IC_50_ value of 37.52 µg/mL. Moreover, different subspecies of *H. arenarium* were investigated for their antioxidant proprierties by Albayrak et al. [21] and the results, expressed as IC_50,_ were: 23.03 µg/mL for subsp. *erzincanicum*, 47.64 µg/mL for subsp. *rubicundum*, 27.32 µg/mL for subsp. *araxinum* and 38.82 µg/mL for subsp. *pseudoplicatum*. Due to different ways of expression, these results are not comparable, and, therefore, further studies are necessary for the evaluation of antioxidant capacity, in correlation with other species/subspecies. Nonetheless, the maximum antioxidant capacity is expressed in all cases in the EtOH 70% (*v*/*v*) extract, which is correlated to the data obtained for phenolic composition of the extracts.

### 2.5. Determination of Total Phenolic Content (TPC) and Total Flavonoid Content (TFC)

**Figure 2:**
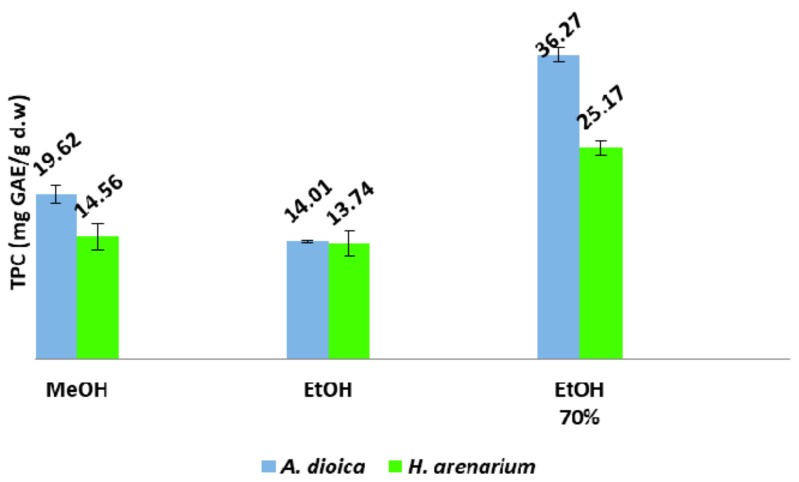
Total phenolic content (TPC) of *H. arenarium* and *A. dioica* flowers.

Besides the identified compounds, many other phenolic compounds are found in the flowers of *H. arenarium* and *A. dioica* and contribute to their antioxidant activity. A comparative overview of the total phenolic and flavonoid contents of the different extracts of the flowers of *H. arenarium* and *A. dioica* species was performed. The total phenolic content (TPC) ranged from 13.74 mg GAE/g d.w. ethanolic extracts of *H. arenarium* to 36.27 mg GAE/g d.w. for 70% (*v*/*v*) ethanolic extract of *A. dioica* extract (Figure 2).

In both cases, the highest values in terms of total phenolic content were obtained by using 70% (*v*/*v*) ethanol as extraction solvent. Additionally, in all cases, values obtained for *A. dioica* extracts were superior. In a study performed by Albayrak et al. [28] on *Helichrysum* sp. from Turkey, a TPC value of 115.76 mg GAE/g of plant extract for methanolic extracts of *H. arenarium* subsp. *aucheri* was obtained. Moreover, Grădinaru et al. [22] found a total phenolic content value of 160.17 mg GAE/g d.w. for a methanolic extract of *H. arenarium* collected from Eastern part of Romania. However, these results are rather hard to be compared with the ones presented herein, due to different extraction solvents, as well as different ways of expressing values. 

**Figure 3:**
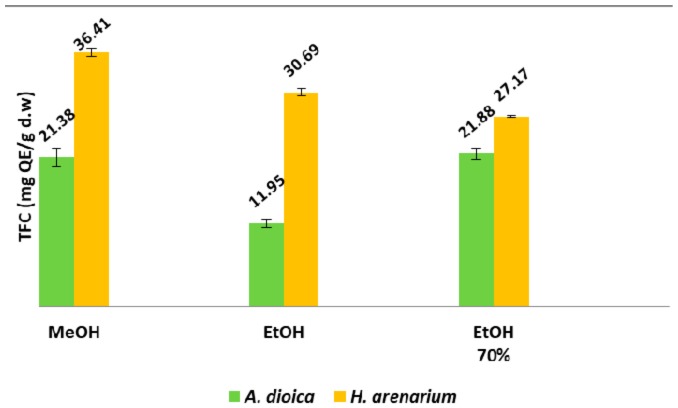
Total flavonoid content (TFC) of *H. arenarium* and *A. dioica* species.

Regarding the total flavonoid content, the highest value was obtained for the methanolic extract of *H. arenarium* (36.41 mg QE/g d.w.), while the lowest values were registered for the ethanolic extract of *A. dioica* (11.95 mg QE/g d.w.) (Figure 3). As a peculiarity, despite different extractive solvents, the TFC values obtained for *H. arenarium* extracts were higher than the ones obtained for *A. dioica* extracts.

### 2.6. Assay of the Antimicrobial Activity

The antibacterial and antifungal activities of *H. arenarium* and *A. dioica* species were tested against a panel of five bacteria and five fungi, selected based on their relevance for public health. Additionally, up-to-date information about the antibacterial and antifungal activities of extracts from *H. arenarium* and *A. dioica* collected from Romania is limited. *Staphylococcus aureus* and *Escherichia coli* were the most sensitive strains towards 70% (*v*/*v*) ethanolic extract of *A. dioica* and methanolic extracts of *H. arenarium* and ethanolic extracts of *H. arenarium,* with similar values of MIC—7.81 mg/mL and MBC—15.62 mg/mL (Table 6 and Table 7).

Additionally, 70% (*v*/*v*) ethanolic extracts of *H. arenarium* showed a good antibacterial activity on the *E. coli* strain (MIC—7.81 mg/mL, and MBC—15.62 mg/mL). Nonetheless, the most resistant strain was *Listeria monocytogenes,* showing the highest results for both MIC—62.5 mg/mL and MBC—125 mg/mL values for ethanolic extracts of *A. dioica*. The antibacterial activity of phenolic compounds has been demonstrated in various studies [29]. As a result, the antibacterial activity of *H. arenarium* and *A. dioica* extracts could be attributed at least in part to phenolic compounds.

Concerning the antifungal activity of the samples (Table 8), *Penicillium fumiculosum* exhibited the highest sensitivity to ethanolic extracts of *H. arenarium* and 70% (*v*/*v*) ethanolic extracts of *H. arenarium* with MIC—7.81 mg/mL and MFC—15.62 mg/mL. Moreover, the strain of *Candida albicans* showed a similar susceptibility to the inhibitory MIC—7.81 mg/mL and fungicidal MFC—15.62 mg/mL of 70% (*v*/*v*) effects of ethanolic extracts of *H. arenarium*. Additionally, the most resistant fungal strains were *Aspergillus flavus* and *Aspergillus niger* towards the methanolic extracts of *A. dioica* with MIC—125 mg/mL and MFC—250 mg/mL, respectively. Overall, *H. arenarium* extracts presented a superior antifungal activity than *A. dioica* samples, for all tested fungal strains. The antimicrobial activity of the studied *Helichrysum* and *Antennaria* species could be at least partially ascribed to the presence of chlorogenic acid. According to Lou et al. [30], chlorogenic acid induced lethal effect on both Gram-positive and Gram-negative bacteria by provoking irreversible permeability changes in the cell membrane, causing the cells to lose the ability to maintain membrane potential.

## 3. Materials and Methods

### 3.1. Plant Material

Flowers of *H. arenarium* were collected from Botoșani county (North Eastern Romania), while flowers of *A. dioica* were collected from Suceava county, (North Eastern Romania). The species were identified by Dr. Ramona Păltinean, and voucher specimens were deposited in the Herbarium of the Department of Pharmaceutical Botany, Faculty of Pharmacy, “Iuliu Hațieganu” University of Medicine and Pharmacy, Cluj-Napoca, Romania (*H. arenarium*—Voucher No. 143.17.3.1 and *A. dioica*—Voucher No. 143.14.1.1).

### 3.2. Extraction Procedure

Dried flowers belonging to the two species were ground to a fine powder. This powder (1.00 g) was mixed with the appropriate solvent (20.00 mL) in a round-bottom flask and stirred for 20 min at 600 rpm on a mechanical stirrer. The solutions were then extracted by sonication for 30 min, at 70 °C, using as solvents methanol (99.98%), ethanol (96%) and 70% *v*/*v* ethanol in water. Subsequently, extracts were filtered and evaporated to dryness under reduced pressure, using a rotary evaporator. The crude extracts were weighed and stored in the refrigerator until they were analyzed. All analyses were performed in triplicate on the three types of extracts and the results were presented as mean ± SD. 

In order to obtain more accurate data on flavonoid glycosides and aglycones concentration, each sample was analyzed before and after acid hydrolysis. Extractive solution (2.00 mL) was treated with 2 M hydrochloric acid (2.00 mL) and a 100 mg/mL ascorbic acid solution (0.20 mL). The mixtures were heated at 80 °C on a water bath for 30 min, ultrasonicated for 15 min, and re-heated for another 30 min at 80 °C. During the heating, 1.00 mL of each solvent was added to the extraction mixture every 10 min, in order to ensure a constant volume. The mixtures were centrifuged at 4000 rpm and the solutions were diluted with distilled water in a 10.00 mL volumetric flask and filtered through a 0.45 µm filter before injection [31]. For the antimicrobial activity, the obtained extracts were evaporated to dryness under reduced pressure and re-suspended in bi-distilled water.

### 3.3. Chemicals

References used for the HPLC-MS analysis were purchased from Sigma Aldrich (St. Louis, MO, USA): Chlorogenic acid, *p*-coumaric acid, caffeic acid, rutin, apigenin, quercetin, isoquercitrin, quercitrin, hyperoside, kaempferol, myricetol, and fisetin, Roth (Karlsruhe, Germany): Ferulic acid, sinapic acid, gentisic acid, gallic acid, patuletin, luteolin or from Dalton (Toronto, ON, Canada): cichoric acid, caftaric acid. HPLC grade solvents, analytical grade acids used for mobile phases and Folin-Ciocâlteu reagent were purchased from Merck (Darmstadt, Germany), together with sodium carbonate, dipotassium hydrogen phosphate, potassium dihydrogen phosphate and aluminium chloride used for antioxidant assays. ABTS (2,2′-azino-bis(3-ethylbenzothiazoline-6-sulfonic acid) diammonium salt) ≥98% purity, potassium peroxodisulfate (≥99% purity), DPPH, and Trolox (6-hydroxy-2,5,7,8-tetramethylchromane-2-carboxylic acid; ≥97% purity) also used in antioxidant tests were purchased from Sigma Aldrich (Schnelldorf, Germany). Gallic acid monohydrate (99.5%) was purchased from Serva, (Heidelberg, Germany).

### 3.4. HPLC-MS Analysis

#### 3.4.1. Apparatus and Chromatographic Conditions

Polyphenols, methoxylated flavones and phytosterols were analyzed and quantified using an HPLC-MS method. The apparatus consisted in an 1100 HPLC Series Agilent Technologies system (Agilent, Santa Clara, CA, USA), equipped with a G1322A degasser, G13311A binary gradient pump, column thermostat, G1313A autosampler and G1316A UV detector. The HPLC system was coupled with an Agilent 1100 mass spectrometer (LC/MSD Ion Trap VL). The chromatographic data were processed using ChemStation and DataAnalysis software from Agilent [25,32,33,34].

#### 3.4.2. HPLC-MS Analysis of Phenolic Compounds

Analysis of phenols was carried out on by injecting 5 µL of the flowers extracts of *H. arenarium* and *A. dioica* on a Zorbax SB-C18 reverse-phase analytical column (100 × 3.0 mm i.d., 3.5 µm particles) and separation of compounds was performed using as a mobile phase a mixture of methanol and acetic acid 0.1% *v*/*v*. The flow rate was set at 1 mL/min and working temperature was 48 °C. The binary gradient that allowed the elution of compounds started with 5% methanol in a linear mode and ended with 42% methanol, for 35 min. The last 3 min of the gradient were set at 42% methanol. Detection of the compounds was performed on UV, at 330 nm until 17.5 min and at 370 nm until the end of the analysis time. The MS was also used for detection of compounds and was operated in a negative mode, using an electrospray ion source. Using these conditions, all polyphenols could be eluted in less than 40 min (Table 9) [25,32,33,34]. 

The specific mass spectra of each polyphenol were recorded in a library and the MS traces/spectra of the compounds that were found in samples were compared with spectra existing in the library. Qualitative analysis of polyphenols was performed based on the MS signal, used for identification of compounds; only compounds identified on basis of their MS spectra were further quantified using the UV signal. Quantitative analyses were performed using the external standard method, with calibration curves of 5 points, ranging between 0.5–50 µg/mL, having a good linearity (*R*^2^ > 0.999). Limit of quantification in this method was 0.5 µg/mL, and the limit of detection was 0.1 µg/mL [25,32,33,34].

#### 3.4.3. HPLC-MS Analysis of Methoxylated Flavones

Analysis of methoxylated flavones was carried out on the same HPLC instrument, using the same analytical column. Mobile phase also consisted in the mixture of 0.1% (*v*/*v*) acetic acid and methanol, but ratios of solvents were changed in order to better perform the separation of compounds. Total duration of the method was less than 10 min and gradient began with 45% methanol and ended at 50% methanol, with a flow rate of 0.9 mL/min. Injection volume was 5 µL and temperature was set at 48 °C. Detection was performed on the same MS/MS system, using an electrospray ionization (ESI) source, in negative mode. Conditions used for detection were set as following: nebulizer pressure at 60 psi, gas (nitrogen) temperature at 325 °C with a flow rate of 12 L/min, and capillary voltage +2500 V. Specific fragments were monitored. Identification was achieved by comparison of retention times and mass spectra for the existing references (Table 10) and compounds identified in samples. Parent ions were detected as forms of the molecules, which have lost a proton. The multiple reaction monitoring mode was used in order to avoid background interferences [33,34].

#### 3.4.4. HPLC-MS Analysis of Phytosterols

Conditions for the analysis of phytosterols were also slightly different from the ones described in the analysis of polyphenols and methoxylated flavones. Same apparatus was used for the separation of compounds. Chromatographic analytical column was the same, but elution of compounds was performed in an isocratic mode, in a mixture of 10:90 (*v*/*v*) of methanol and acetonitrile. Flow rate was set at 1 mL/min, chromatographic system was operated at 40 °C and the injection volume was 5 µL. For detection of compounds, the same mass spectrometer was used, with an atmospheric pressure chemical ionization (APCI) interface, in a positive mode. Conditions were set as following: gas temperature (nitrogen) 325 °C at a flow rate of 7 L/min, nebulizer pressure 60 psi and capillary voltage −4000 V. Identification of compounds was performed on the same basis as the one performed for methoxylated flavones, using the multiple reactions monitoring analysis model (Table 11) [32]. 

### 3.5. Determination of Total Phenolic Content

The TPC was determined using the Folin–Ciocâlteu method. For a high throughput of samples, a SPECTROstar Nano Multi—Detection Microplate Reader with 96-well plates (BMG Labtech, Ortenberg, Germany) was used. Briefly, a mixture solution consisting of 20 µL of extract, 100 µL of Folin-Ciocâlteu reagent and 80 µL of sodium carbonate (Na_2_CO_3_, 7.5% *w*/*v*) was homogenized and incubated at room temperature in the dark for 30 min. Afterwards, the absorbance of the samples was measured at 760 nm. Gallic acid was used as a reference standard, and the TPC was expressed as gallic acid equivalents (GAE) in mg/g dry weight (d.w.) of plant material [35,36]. 

### 3.6. Determination of Total Flavonoid Content

The total flavonoid content (TFC) was calculated and expressed as quercetin equivalents using a method previously described by Mocan et al. [37,38]. Briefly, a 100 µL aliquot of 2% AlCl_3_ aqueous solution was mixed with 100 µL of sample. After an incubation time of 15 min, the absorbance of the sample was measured at 420 nm. Quercetin was used as a reference standard, and the TFC was expressed as quercetin equivalents (QE) in mg/g dry weight (d.w.) of plant material. 

### 3.7. Antioxidant Activity Assays

#### 3.7.1. TEAC Assay

The TEAC of the different *Helichrysum* and *Antennaria* extracts against the stable synthetic ABTS radical cation was tested using the method described by Martinez et al. [39] and Savran et al. [40]. A Trolox calibration curve was plotted as a function of the percentage of ABTS radical cation scavenging activity, and the results were expressed as milligrams of trolox equivalents (TE) per milliliter of herbal extract (mg TE/mL).

#### 3.7.2. DPPH Assay

The antioxidant capacity of the investigated samples against the DPPH radical was tested using the method previously described by Martins et al. and Mocan et al. [41,42]. A Trolox calibration curve was plotted as a function of DPPH consumption, and the results were expressed as milligrams of trolox equivalents (TE) per milliliter of herbal extract (mg TE/mL).

### 3.8. Assay of Antimicrobial Activity

#### 3.8.1. Bacteria and Culture Conditions

For this bioassay, five aerobic bacterial strains were used, *Staphylococcus aureus* (ATCC 49444), *Bacillus cereus* (ATCC 11778), *Listeria monocytogenes* (ATCC 19114), *Salmonella typhimurium* (ATCC 14028), and *Escherichia coli* (ATCC 25922). All of the tested microorganisms were obtained from the Laboratory of Food Biotechnology, Life Sciences Institute, University of Agricultural Sciences and Veterinary Medicine, Cluj Napoca, Romania. The bacteria were cultured on Muller-Hinton Agar and stored at 4 °C and subcultured once a month. 

The modified microdilution technique was used to evaluate antimicrobial activity as previously reported by Mocan et al. [29,38]. Bacterial species were cultured overnight at 37 °C in Tryptic Soy Broth (TSB) medium. The bacterial cell suspensions were adjusted with sterile saline to a concentration of approximately 3 × 10^5^ CFU/mL in a final volume of 100 µL per well. The inoculum was stored at +4 °C for further use. Dilutions of the inoculum were cultured on solid Muller–Hinton (MH) for bacteria to verify the absence of contamination and to check the validity of the inoculum. Determinations of minimum inhibitory concentrations (MICs) were performed by a serial dilution technique using 96-well microtitre plates. The concentration range of extracts was 1000–0.03 mg/mL. Different solvent dilutions of ethanol: methanol extracts were carried out over the wells containing 100 µL of Tryptic Soy Broth (TSB) and afterwards, 10 µL of inoculum was added to all the wells. The microplates were incubated for 24–48 h at 37 °C. The MIC of the samples was detected following the addition of 20 µL (0.2 mg/mL) of resazurin solution to each well, and the plates were incubated 2 h at 37 °C. A change from blue to pink indicates reduction of resazurin and, therefore, bacterial growth. The MIC was defined as the lowest drug concentration that prevented this color change. The minimum bactericidal concentrations (MBCs) were determined by serial subcultivation of a 2 µL into microtitre plates containing 100 µL of broth per well and further incubation for 48 h at 37 °C. The lowest concentration with no visible growth was defined as MBC, indicating 99.5% killing of the original inoculum. Streptomycin (Sigma P 7794) (0.05–3 mg/mL) was used as positive control. Water was used as negative control. 

#### 3.8.2. Antifungal Activity

To investigate the antifungal activities, the following fungi were used: *Aspergillus flavus* (ATCC 9643), *Aspergillus niger* (ATCC 6275), *Candida albicans* (ATCC 10231), *Candida parapsilosis* (ATCC 22019) and *Penicillium funiculosum* (ATCC 56755). These fungi were obtained from the Laboratory of Food Biotechnology, Life Sciences Institute, University of Agricultural Sciences and Veterinary Medicine, Cluj-Napoca, Romania. Cultures were maintained on malt agar at 4 °C and subcultured every month. Spore suspension (1.0 × 10^5^) was obtained by washing agar plates with sterile solution containing (0.85% saline, 0.1% Tween 80 (*v*/*v*), then added to each well to a final volume of 100 µL. Inocula were screened for contamination by culturing on a solid medium. The minimum inhibitory (MIC) and minimum fungicidal (MFC) concentrations assays were performed using the microdilution method by preparing a serial of dilutions in 96-well microtiter plates. The extracts were diluted in 0.85% saline (10 mg/mL), then added to microplates containing Broth Malt medium with inoculum and incubated for 72 h at 28 °C on a rotary shaker. The lowest concentrations without visible growth (at the binocular microscope) were defined as minimal inhibitory concentrations (MICs). The fungicidal concentrations (MFCs) were determined by serial sub-cultivation of 2 µL of tested extracts dissolved in medium and inoculated for 72 h, into microtiter plates containing 100 µL of broth per well and further incubation 72 h at 28 °C. The lowest concentration with no visible growth was defined as MFC indicating 99.5% killing of the original inoculum. The fungicide fluconazole was used as positive control (1–3500 µg/mL). All experiments were performed in duplicate and repeated thrice [29].

### 3.9. Statistical Analysis

The samples have been analyzed in triplicate; the average and the relative SD have been calculated using the Excel software package (Microsoft, Redmond, WA, USA).

## 4. Conclusions

An in-depth chemical composition of several classes of bioactive compounds revealed that traditionally used Romanian *H. arenarium* and *A. dioica* represent important sources of bioactive compounds with potent in vitro activities. According to the obtained results, *H. arenarium* and *A. dioica* 70% EtOH extracts represent important sources of chlorogenic acid and flavonoids. Moreover, the biological properties of these species indicate their potential usage in oxidative stress related disorders. Particularly, even though the investigated species are traditionally used as herbal remedies with antimicrobial potential, the results obtained for the current investigated samples are modest. However, further studies are necessary in order to elucidate the mechanisms of in vivo antioxidant and microbial inhibition action, bioavailability and involved metabolic pathways.

## Figures and Tables

**Table 1:** The phenolic compounds identified in the flowers of *H. arenarium* and *A. dioica* extracts before acid hydrolysis (mg/100 g d.w.).

Phenolic Compound	*H. arenarium* (L.) Moench.			*A. dioica* (L.) Gaertn.		
	MeOH	EtOH	EtOH 70%	MeOH	EtOH	EtOH 70%
Chlorogenic acid	177.70 ± 8.88	111.58 ± 5.57	340.95 ± 17.04	434.18 ± 21.70	84.59 ± 4.22	502.70 ± 25.11
*p*-Coumaric acid	NF	1.18 ± 0.05	NF	17.13 ± 0.85	NF	NF
Isoquercitrin	70.18 ± 3.50	57.45 ± 2.87	85.55 ± 4.27	NF	NF	NF
Quercitrin	438.33 ± 21.91	409.09 ± 20.45	424.28 ± 21.21	444.48 ± 22.22	147.64 ± 7.38	240.41 ± 12.02
Luteolin	9.34 ± 0.46	8.66 ± 0.43	9.98 ± 0.49	NF	NF	NF
Kaempferol	7.61 ± 0.38	7.31 ± 0.36	7.16 ± 0.35	45.67 ± 2.28	15.64 ± 0.78	32.08 ± 1.60
Apigenin	66.96 ± 3.34	63.85 ± 3.19	62.99 ± 3.14	11.42 ± 0.57	4.27 ± 0.21	7.43 ± 0.37

Note: Values represent the mean ± SD (standard deviation); NF = not found, below the limit of quantification.

**Table 2:** The phenolic compounds identified in the flowers of *H. arenarium* and *A. dioica* extracts after acid hydrolysis (mg/100 g d.w.).

Phenolic Compound	*H. arenarium* (L.) Moench.			*A. dioica* (L.) Gaertn.		
	MeOH	EtOH	EtOH 70%	MeOH	EtOH	EtOH 70%
Chlorogenic acid	73.63 ± 3.68	64.07 ± 3.20	224.39 ± 11.21	38.43 ± 1.92	27.97 ± 1.39	246.55 ± 12.32
*p*-Coumaric acid	2.65 ± 0.13	2.16 ± 0.10	4.40 ± 0.22	3.17 ± 0.15	2.41 ± 0.12	13.13 ± 0.65
Ferulic acid	NF	NF	4.08 ± 0.20	NF	NF	NF
Isoquercitrin	NF	NF	13.89 ± 0.69	NF	NF	NF
Quercitrin	7.25 ± 0.36	9.19 ± 0.45	15.85 ± 0.79	63.04 ± 3.15	69.38 ± 3.46	89.65 ± 4.48
Quercetol	23.31 ± 1.16	20.68 ± 1.03	26.16 ± 1.30	12.17 ± 0.60	9.34 ± 0.46	19.66 ± 0.98
Luteolin	5.76 ± 0.28	7.19 ± 0.35	9.09 ± 0.45	128.15 ± 6.40	104.70 ± 5.23	183.52 ± 9.17
Kaempferol	181.23 ± 9.06	175.63 ± 8.78	179.28 ± 8.96	1.95 ± 0.09	0.85 ± 0.04	1.89 ± 0.09
Apigenin	69.79 ± 3.48	61.87 ± 3.09	59.07 ± 2.95	20.79 ± 1.03	16.71 ± 0.83	24.31 ± 1.21

Note: Values represent the mean ± SD (standard deviation); NF = not found, below the limit of quantification.

**Table 3:** The methoxylated flavones identified in the flowers of *H. arenarium* and *A. dioica* extract (mg/100 g d.w.).

Methoxylated Flavone	*Helichrysum arenarium*			*Antennaria dioica*		
	MeOH	EtOH	EtOH 70%	MeOH	EtOH	EtOH 70%
Hispidulin	0.70 ± 0.03	0.68 ± 0.03	0.68 ± 0.01	0.29 ± 0.01	0.22 ± 0.01	0.33 ± 0.01

Note: Values represent the mean ± SD (standard deviation).

**Table 4:** The sterolic compounds identified in the flowers of *H. arenarium* and *A. dioica* extracts (mg/100 g d.w.).

Sterolic Compound	*Helichrysum arenarium*			*Antennaria dioica*		
	MeOH	EtOH	EtOH 70%	MeOH	EtOH	EtOH 70%
Campesterol	1.04 ± 0.05	0.82 ± 0.04	0.80 ± 0.03	1.36 ± 0.06	0.95 ± 0.04	1.50 ± 0.07
Stigmasterol	6.17 ± 0.30	4.37 ± 0.21	5.40 ± 0.21	4.72 ± 0.23	2.90 ± 0.14	4.79 ± 0.23
β-Sitosterol	37.04 ± 1.85	26.21 ± 1.31	26.21 ± 1.04	63.77 ± 3.18	45.92 ± 2.29	67.58 ± 3.37

Note: Values represent the mean ± SD (standard deviation).

**Table 5:** Antioxidant capacity parameters obtained using several methods for studied samples.

Samples		TEAC (mg TE/mL)	DPPH (mg TE/mL)
***A. dioica***	**MeOH**	3.89 ± 0.01	13.44 ± 1.65
	**EtOH**	2.65 ± 0.01	5.89 ± 1.23
	**EtOH 70%**	5.71 ± 0.01	15.21 ± 1.97
***H. arenarium***	**MeOH**	4.04 ± 0.01	4.91 ± 1.90
	**EtOH**	3.71 ± 0.01	7.21 ± 2.81
	**EtOH 70%**	5.82 ± 0.02	17.88 ± 7.20

Note: Values represent the mean ± SD (standard deviation).

**Table 6:** Minimum inhibitory concentration—MIC (mg/mL) of *H. arenarium* and *A. dioica* extracts and standard antibiotic.

Samples		*Staphylococcus aureus (ATCC 49444)*	*Bacillus cereus (ATCC 11778)*	*Listeria monocytogenes (ATCC 19114)*	*Salmonella typhimurium (ATCC 14028)*	*Escherichia coli (ATCC 25922)*
***A. dioica***	**MeOH**	62.5	62.5	31.25	31.25	15.62
	**EtOH**	15.62	31.25	62.5	31.25	15.62
	**EtOH 70%**	7.81	31.25	31.25	31.25	7.81
***H. arenarium***	**MeOH**	7.81	31.25	31.25	62.5	7.81
	**EtOH**	7.81	15.62	15.62	62.5	7.81
	**EtOH 70%**	15.62	15.62	31.25	31.25	7.81
**Streptomycin**		0.03	0.015	0.015	0.06	0.12

**Table 7:** Minimum bactericidal concentration—MBC (mg/mL) of *H. arenarium* and *A. dioica* extracts and standard antibiotic.

Samples		*Staphylococcus aureus (ATCC 49444)*	*Bacillus cereus (ATCC 11778)*	*Listeria monocytogenes (ATCC 19114)*	*Salmonella typhimurium (ATCC 14028)*	*Escherichia coli (ATCC 25922)*
***A.dioica***	**MeOH**	125	125	62.5	62.5	31.25
	**EtOH**	31.25	62.5	125	62.5	31.25
	**EtOH 70%**	15.62	62.5	62.5	62.5	15.62
***H. arenarium***	**MeOH**	15.62	62.5	62.5	125	15.62
	**EtOH**	15.62	31.25	31.25	125	15.62
	**EtOH 70%**	31.25	31.25	62.5	62.5	15.62
**Streptomycin**		0.06	0.030	0.030	0.12	0.24

**Table 8:** Minimum inhibitory concentration MIC (mg/mL) and minimum fungicidal concentration MFC (mg/mL) of *H. arenarium* and *A. dioica* extracts and standard antibiotic.

	*A. dioica*						*H. arenarium*						Fluconazole	
	MeOH		EtOH		EtOH 70%		MeOH		EtOH		EtOH 70%			
	MIC	MFC	MIC	MFC	MIC	MFC	MIC	MFC	MIC	MFC	MIC	MFC	MIC	MFC
***Aspergillus flavus***	125	250	62.5	125	62.5	125	62.5	125	31.25	62.5	31.25	62.5	0.15	0.3
***Aspergillus niger***	125	250	62.5	125	31.25	62.5	62.5	125	31.25	62.5	31.25	62.5	0.15	0.3
***Candida albicans***	31.25	62.5	31.25	62.5	15.62	31.25	31.25	62.5	15.62	31.25	7.81	15.62	0.10	0.2
***Candida parapsilosis***	62.5	125	31.25	62.5	31.25	62.5	31.25	62.5	15.62	31.25	15.62	31.25	0.10	0.2
***Penicillium fumiculosum***	15.62	31.25	15.62	31.25	15.62	31.25	15.62	31.25	7.81	15.62	7.81	15.62	0.15	0.3

**Table 9:** Retention times (RT) and values of *m*/*z* for the tested polyphenolic compounds.

Peak No.	Phenolic Compounds	*m*/*z*	RT ± SD (min)
**1**	Caftaric acid	311	3.54 ± 0.05
**2**	Gentisic acid	179	3.52 ± 0.04
**3**	Caffeic acid	179	5.60 ± 0.04
**4**	Chlorogenic acid	353	5.62 ± 0.05
**5**	*p*-Coumaric acid	163	9.48 ± 0.08
**6**	Ferulic acid	193	12.8 ± 0.10
**7**	Sinapic acid	223	15.00 ± 0.10
**8**	Cichoric acid	473	15.96 ± 0.13
**9**	Hyperoside	463	18.60 ± 0.12
**10**	Isoquercitrin	463	19.60 ± 0.10
**11**	Rutin	609	20.20 ± 0.15
**12**	Myricetin	317	21.13 ± 0.12
**13**	Fisetin	285	22.91 ± 0.15
**14**	Quercitrin	447	23.64 ± 0.13
**15**	Quercetin	301	26.80 ± 0.15
**16**	Patuletin	331	29.41 ± 0.12
**17**	Luteolin	285	29.10 ± 0.19
**18**	Kaempferol	285	32.48 ± 0.17
**19**	Apigenin	279	33.10 ± 0.15

Note: Values represent the mean ± SD (standard deviation).

**Table 10:** Retention times (RT) and specific ions of the tested methoxylated flavones.

Compound	Retention Time (min)	M	M − H^−^	Ions/Fragments
Jaceosidin	2.9	330.3	329.3	314
Hispidulin	4.2	300.2	299.2	284
Eupalitin	7.05	344.3	343.3	328
Eupatorin	7.6	344.3	343.3	328
Casticin	8.05	374.3	373.3	358
Acacetin	9.8	284.3	283.3	268

**Table 11:** Retention times (RT) and specific ions of the phytosterols.

Phytosterol	Retention Time (min)	M	M − H_2_O	M − H_2_O + H^+^
Ergosterol	2.6	396	378	379
Brassicasterol	3.3	398	380	381
Stigmasterol	4.0	412	394	395
Campesterol	4.0	400	382	383
Sitosterol	4.6	414	396	397
